# Efficacy of Polynucleotides in Periorbital Rejuvenation: A Systematic Review

**DOI:** 10.1093/asjof/ojag200

**Published:** 2026-10-10

**Authors:** Rhea Ahuja, Samar Khalil, Hassan I Galadari, Stefania Guida

## Abstract

Periorbital aging is characterized by dynamic wrinkles, volume loss, and dermal quality decline. Injectable polynucleotides (PNs) have emerged as biostimulatory agents for periorbital rejuvenation, and this review evaluates their efficacy and safety for this indication. The review was conducted per PRISMA 2020 guidelines and registered on PROSPERO (CRD420261288052). MEDLINE and Embase were searched from inception to January 30, 2026, for studies of adults receiving injectable PN for periorbital rejuvenation; no meta-analysis was performed given anticipated heterogeneity. Six studies (5 randomized split-face trials, 1 prospective case series; *n* = 350) met inclusion criteria, all conducted in Korean and Thai cohorts with Fitzpatrick III-V skin and predominantly middle-aged women (80%-100% female). Polynucleotide produced consistent short-term improvements in crow's feet severity and dermal quality metrics, with 40% to 70% of treated sides achieving at least a 1-grade Crow's Feet Grading Scale improvement at 12 weeks, reflecting cross-trial heterogeneity rather than a single pooled effect. In 2 trials (*n* = 30 and *n* = 215), PN was noninferior to polycaprolactone fillers for crow's feet correction; in 1 trial (*n* = 30), PN showed favorable biophysical outcomes relative to hyaluronic acid. Safety was favorable throughout, but no study followed patients beyond 6 months. In these East Asian, predominantly female cohorts, PN demonstrates short-term efficacy and a favorable safety profile through 6 months for periorbital rejuvenation. Given the small number of studies, heterogeneity, and lack of longer-term follow-up, findings should be regarded as preliminary. Generalization beyond the populations and timeframes studied awaits larger, longer-term, ethnically diverse trials with direct comparison to established modalities.

**Level of Evidence**: 4 (Therapeutic) 
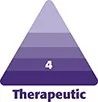

Periorbital aging represents one of the most noticeable signs of facial senescence, characterized by volume loss, skin texture deterioration, and dynamic wrinkles, particularly in the tear trough and periorbital (crow's feet) regions.^1^ The periocular area is particularly vulnerable to aging because of thinner, more delicate dermis and increased dynamic muscle activity.^2^ Conventional approaches to periorbital rejuvenation have relied on hyaluronic acid (HA) fillers and autologous fat grafting, which provide mechanical volume restoration but may not address underlying dermal quality or collagen depletion.^3^

Recent advances in regenerative dermatology have introduced injectable polynucleotides (PNs) as biostimulatory agents for aesthetic skin rejuvenation. Polynucleotides are high-molecular-weight salmon DNA fragments (typically over 1500 kDa) that stimulate endogenous collagen synthesis, improve dermal hydration, and promote tissue remodeling through fibroblast activation and growth factor production.^4^ Beyond fibroblast stimulation, PN exerts broader biologic effects relevant to dermal aging: it supports extracellular matrix remodeling and balanced collagen homeostasis through regulation of matrix metalloproteinases and their tissue inhibitors, promotes local angiogenesis and microcirculatory improvement, exerts anti-inflammatory and antioxidant effects that limit aging-related tissue damage (Figure 1).^4,5^ Polydeoxyribonucleotides (PDRNs), although chemically related, consist of low-molecular-weight DNA fragments (typically 50-1500 kDa) that act through pharmacologic activation of adenosine A_2_A receptors, resulting in increased angiogenesis, upregulation of vascular endothelial growth factor, and modulation of inflammatory responses, which collectively facilitate tissue repair and wound healing.^6^ Consequently, although PNs are utilized as injectable agents for skin rejuvenation, PDRN is primarily indicated for regenerative and wound-healing applications. Accordingly, PDRN-based products are not included in this review. These distinctions are maintained throughout this review: the term “polynucleotide” is used exclusively to denote the high-molecular-weight, fibroblast-biostimulatory agents described above, distinct from PDRN's receptor-mediated pharmacologic activity, and the 2 agents should not be treated as interchangeable when interpreting efficacy data. A recent systematic review examined PN across the broader field of esthetic medicine; the present review instead focuses specifically on the periorbital region, allowing a more detailed evaluation of evidence for this indication.^7^

**Figure 1.**
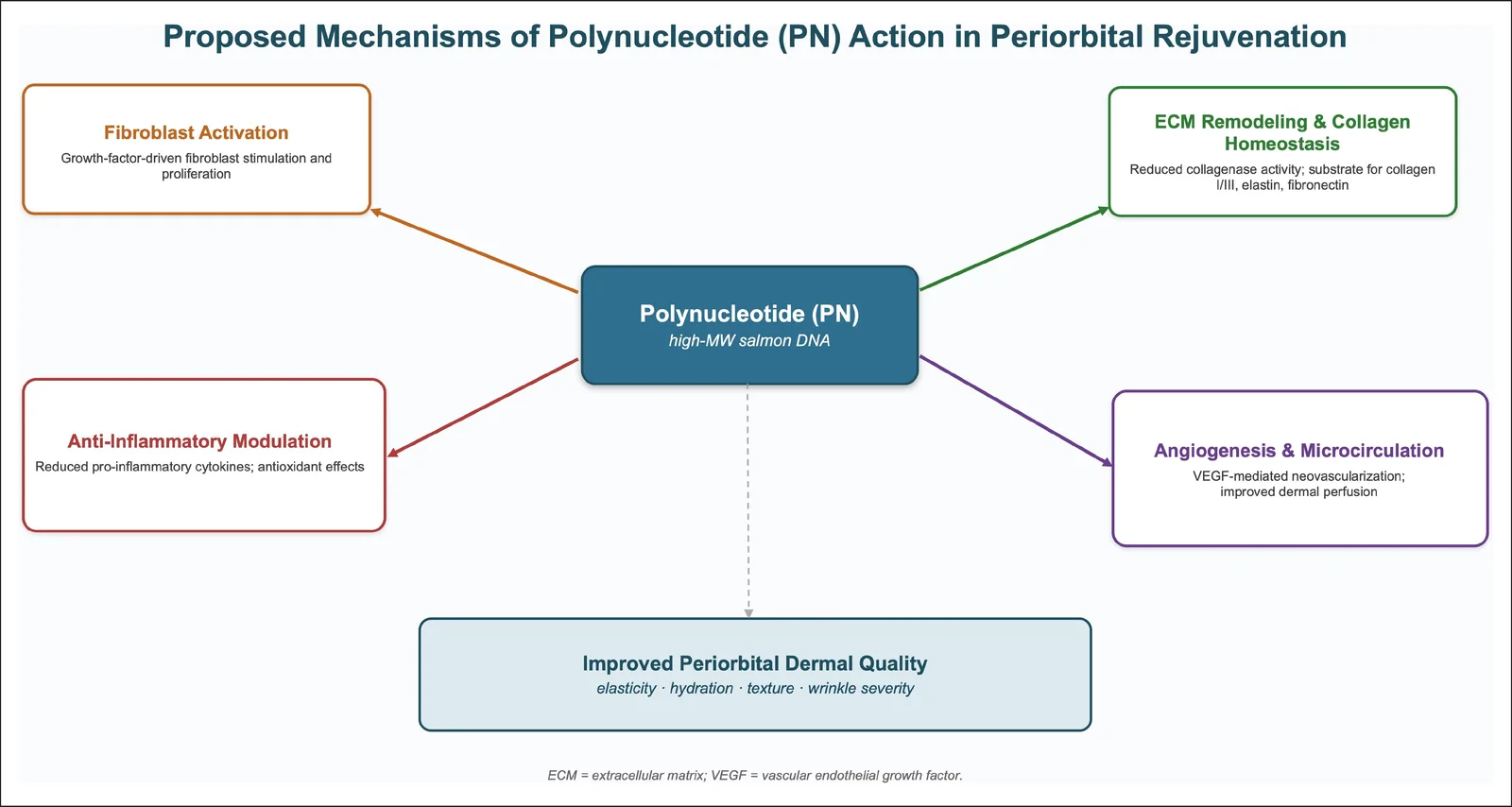
Conceptual illustration summarizing the proposed biologic mechanisms of action of polynucleotides in the periorbital dermis and their relationship to improvements in periorbital dermal quality. ECM, extracellular matrix; MW, molecular weight; PN, polynucleotide; VEGF, vascular endothelial growth factor.

The periocular region also presents distinct anatomical considerations relevant to injectable regenerative therapies. The eyelid and infraorbital dermis are markedly thinner than facial skin elsewhere (0.3-0.6 mm over the lower eyelid compared with over 1 mm over the malar cheek), overlying a thin orbicularis oculi muscle and minimal subcutaneous fat.^2^ The orbital septum forms a key boundary separating the preseptal space from the postseptal orbital fat compartments, and its integrity and laxity influence both the clinical presentation of periorbital aging (fat pseudoherniation, tear trough deformity) and the safety of injectable placement. Retaining ligaments, including the orbicularis retaining ligament and the tear trough ligament, anchor the skin–muscle complex to the underlying periosteum, and their attenuation with age contributes to the visible tear trough groove. Adjacent soft-tissue compartments, including the superficial and deep medial cheek fat and the suborbicularis oculi fat, further modulate the contour changes seen with aging.^2^ These features underlie the rationale for superficial, dermal-plane PN injection, which targets the thin overlying dermis rather than the deeper compartments addressed by volumizing fillers, and help explain the differing profiles of PN relative to deeper-plane HA or fat grafting.^8^

Although early clinical observations suggest benefits in tear trough correction, crow's feet reduction, and overall periorbital skin quality, current evidence remains limited and heterogeneous, with few comparative studies against established treatments such as HA fillers.^9,10^ Furthermore, data specific to periorbital application, injection techniques, optimal dosing regimens, and comparative efficacy against other aesthetic modalities remain incompletely characterized. A recent systematic review of PN across broader aesthetic medicine indications highlighted this same evidence gap but did not focus specifically on the periorbital region, underscoring the need for the present indication-specific review.^7^

Given the growing use of PNs for periocular rejuvenation, a systematic evaluation of their clinical efficacy and comparison with alternative modalities, such as HA fillers, microneedling, and energy-based devices, is needed to guide clinical decision making. This review therefore aims to assess the efficacy of PN injections for periorbital rejuvenation—specifically crow's feet reduction, tear trough improvement, and enhancement of periocular skin quality—while also analyzing treatment protocols and evaluating the associated safety profile.

## METHODS

This systematic review followed PRISMA 2020 guidelines.^11,12^ Detailed methodology was published in PROSPERO (CRD420261288052). A completed PRISMA 2020 checklist is provided in Supplemental Table 1. We searched PubMed (MEDLINE) and Embase from database inception to January 30, 2026, using terms for PNs (“polynucleotide” OR “polynucleotides” OR “PN” OR “salmon DNA”) AND periorbital anatomy (“peri-orbital” OR “periocular” OR “tear trough” OR “crow's feet”) (Supplemental Table 2). MEDLINE and Embase were selected as they provide the broadest indexing of peer-reviewed biomedical literature on this topic; Cochrane CENTRAL and regional/Asian-language databases (eg, KoreaMed) were not searched, which is acknowledged as a limitation given the predominance of Korean- and Thai-authored studies in this literature (see Limitations). Included were English-language studies (randomized controlled trials [RCTs], controlled/noncontrolled trials, cohorts, case series ≥5 patients) of adults (≥18 years) receiving injectable PNs for periorbital rejuvenation (tear trough, crow's feet, and skin quality), excluding reviews, commentaries, animal/in vitro studies, and studies with nonperiorbital focus only.

The search was conducted by (R.A.). Two reviewers (R.A. and S.K.) independently screened titles/abstracts and full texts, extracted data (study/participant characteristics, interventions, and outcomes), and assessed bias (RoB 2 for RCTs; ROBINS-I for observational studies); disagreements at any stage were resolved by discussion and, where necessary, adjudication by a third author (H.G.).^13,14^ Primary outcomes were periorbital improvements (tear trough volume/contour, crow's feet severity through validated scales, skin quality metrics like elasticity/hydration through Cutometer [Courage + Khazaka electronic GmbH, Köln, Germany]/Antera 3D [Miravex Limited, Dublin, Ireland]). Secondary outcomes included adverse events (AEs), satisfaction, and treatment parameters. Data were synthesized narratively because of heterogeneity; no meta-analysis was performed.

## RESULTS

The database search identified 42 records from MEDLINE and Embase, of which 17 duplicates were removed, leaving 25 records for title/abstract screening. Of these, 15 were excluded, and 10 reports were sought for retrieval; all 10 were retrieved and assessed for eligibility. Following full-text assessment, 4 were excluded (an in vitro study, a commentary on an included article, a survey among dermatologists, and a PDRN-based study of the tear trough), leaving 6 studies for inclusion in the qualitative synthesis. This process is summarized in the PRISMA flow diagram (Figure 2).

**Figure 2.**
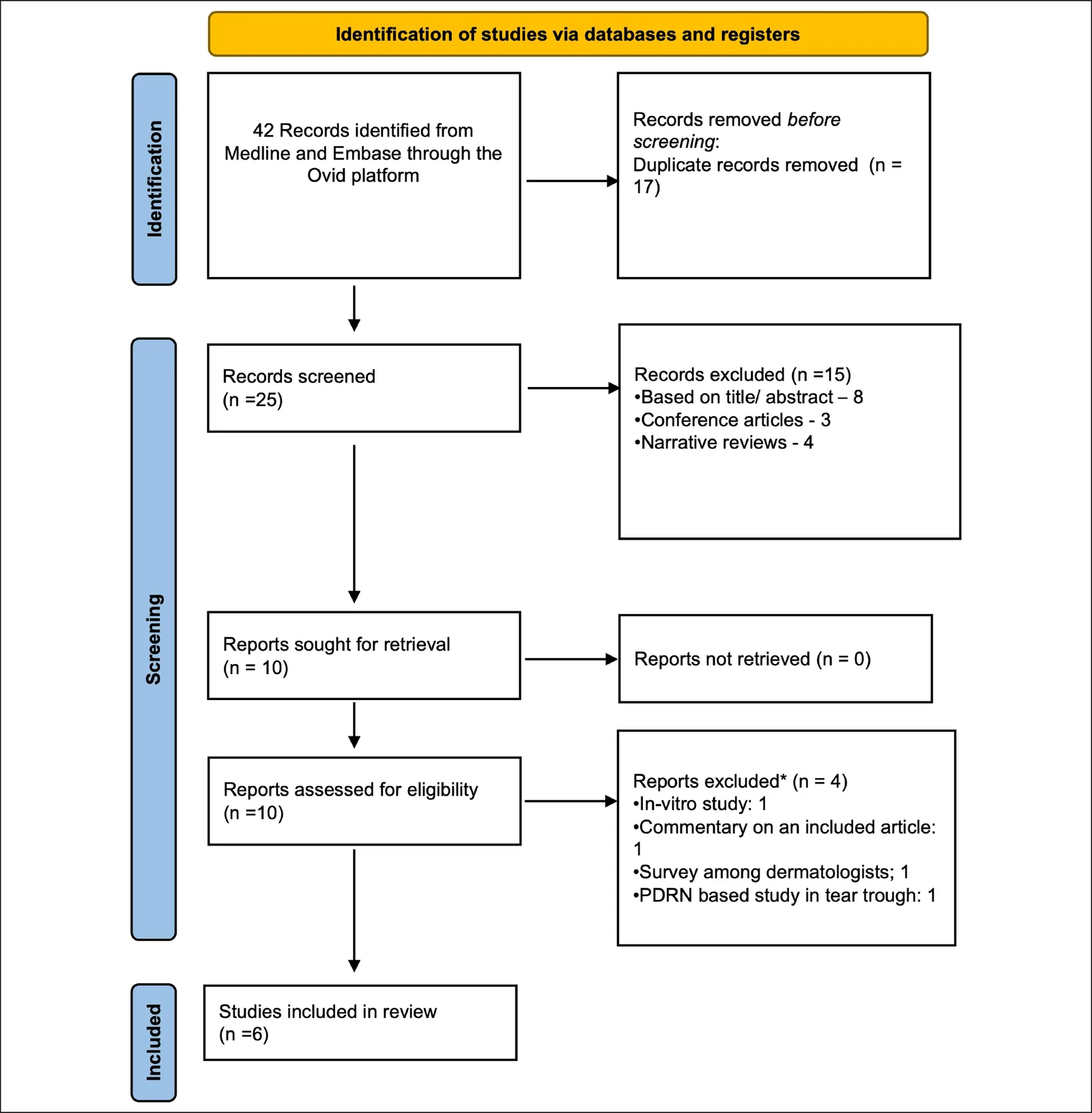
Preferred Reporting Items for Systematic Review and Meta-Analyses (PRISMA) flowchart for the included studies retrieval process. PDRNs, polydeoxyribonucleotides.

Across the 6 included studies, 5 were randomized split-face trials, and 1 was a prospective case series, enrolling a total of 350 adults undergoing periorbital rejuvenation with PN-based injectables. Most participants were middle-aged women; mean ages between the early 40 seconds and mid-50 seconds, and female representation was 80% to 100% across all trials.^9,10,15-18^ All studies were conducted in Asian populations, predominantly Korean and Thai for the RCTs.^9,10,15-18^ Polynucleotide formulations were fairly homogeneous in concentration (20 mg/mL, where specified) and included Rejuran (PharmaResearch Co., Ltd, Seoul, South Korea) or Rejuran i, HP Vitaran/HP cell Vitaran (BR Pharm Co., Ltd, Wonju, South Korea); polycaprolactone (PCL) fillers served as active comparators in 2 RCTs, and HA filler in 1 trial.^8,15,16^ The injection protocols have been outlined in Table 1.

**Table 1.** Summary of Study and Patient Characteristics

Author (year)	Country	Study type	Sample size (enrolled/completed)	Mean age (years)	Sex (% female)	Area studied	Type of intervention	Treatment regimen	Outcome parameters	Follow-up time points	Follow-up duration
Jeong et al (2020)^15^	South Korea	RCT, split-face, patient/evaluator-blinded	30/29	>19 years (NR)	NR	Crow’s feet	PCL (DLMR01) vs PN (Rejuran); 1.0 mL/side, linear threading	3 sessions, 2-week interval	CFGS, GAIS, PRIMOS 3D (Ra, *R*_max_, wrinkle volume)	2, 4, and 12 weeks	12 weeks
Choi et al (2025)^16^	South Korea	RCT, split-face, participant/evaluator-blinded	218/215	52.3 ± 7.0	84.2	Crow's feet	PCL (DLMR01) vs PN (Rejuran); 0.93 ± 0.05 mL/side, linear threading	1 session	CFGS, GAIS	2, 4, and 12 weeks	12 weeks
Lee et al 2022)^9^	South Korea	RCT, double-blind, split-face	30/27	48.8 (37-62)	100	Periorbital (crow’s feet and infraorbital)	PN (Rejuran) vs HA (Yvoire-Hydro); 1.0 mL/side, serial puncture (0.02 mL/0.5-1 cm grid)	4 sessions, 2-week interval	VAS, GAIS, Cutometer (R2, R5, R7), Corneometer, Antera 3D (roughness, pores), skin scanner	10, 16, and 28 weeks	28 weeks
Yogya et al (2022)^17^	Thailand	RCT, split-face	30/29	42.9 ± 6.7	100	Periorbital wrinkles	RFMN alone vs RFMN + topical PN	3 sessions, 2-week intervals	Antera 3D (indentation, max depth), self-evaluation, Cutometer elasticity	2 weeks, 1, 2, 3, and 6 months	6 months
Kim et al (2022)^10^	South Korea	Prospective case series	30/28	55.7	90	Crow's feet	PN (HP cell Vitaran I); 0.5 mL/eye, serial puncture, 4 sessions	4 sessions, 2-week intervals	CFGS, GAIS, Antera 3D (wrinkle, texture, pore, depression, melanin, Hb)	8, 10, and 18 weeks	18 weeks
Jeon et al (2025)^18^	South Korea	RCT, split-face, patient-blinded	15/15	43.9 ± 6.5	80	Periorbital/crow’s feet	Ionization-adjusted PN (HP Vitaran I) vs conventional PN (Rejuran i); 1.0 mL/side, serial puncture	4 sessions, 2-week interval	VAS pain, CFGS, GAIS	2, 4, and 12 weeks	12 weeks

CFGS, Crow's Feet Grading Scale; GAIS, Global Aesthetic Improvement Scale; HA, hyaluronic acid; Hb, hemoglobin; NR, not reported; PCL, polycaprolactone; PN, polynucleotide; RCT, randomized controlled trial; RFMN, radiofrequency microneedling; VAS, visual analog scale.

The efficacy was primarily evaluated with the Crow's Feet Grading Scale (CFGS) and Global Aesthetic Improvement Scale (GAIS), supplemented by objective instrumental measurements. Three studies used the Antera 3D imaging system to quantify wrinkle indentation, depth, texture, pore size, depression, melanin, and hemoglobin indices 1 used PRIMOS 3-dimensional profilometry for Ra and related roughness parameters; 1 combined Cutometer-based elasticity (R2, R5, R7) and Corneometer (Courage + Khazaka electronic GmbH, Köln, Germany) hydration measures, together with high-frequency ultrasound for dermal thickness.^5,9,10,12,17^ Follow-up time points are discussed in Table 1.

The efficacy results across the 6 studies are summarized in Table 2. In Kim et al, a 30-patient prospective case series on PN filler for crow's feet, the mean CFGS improved from 2.77 at baseline to 1.84 at 18 weeks, with highly significant reductions evident from Week 8 onwards and maintained through the final visit. Objective Antera parameters mirrored these findings: wrinkle, texture, depression, and pore indices all improved significantly compared with baseline.^10^

**Table 2.** Summary of Efficacy Results Across Included Studies

Study (author, year) Sample size (*n*, enrolled/completed)	Design and comparator	Primary efficacy outcome	Key results (primary)	Key secondary outcomes	Effect size/significance
Jeong et al (2020)^15^ *n* = 30/29	RCT split-face PCL vs PN	CFGS improvement ≥1 grade at 12 weeks (rest)	PCL: 48.3% PN: 41.4%	GAIS: similar PRIMOS Ra: no difference	NS (*P* = .56)
Choi et al (2025)^16^ *n* = 218/215	RCT split-face PCL vs PN	CFGS improvement ≥1 grade at 12 weeks (rest)	PCL: 70.2% PN: 61.4%	GAIS: similar CFGS expression: similar	PCL noninferior (+8.8%, *P* < .05)
Lee et al (2020) *n* = 30/27	RCT split-face PN vs HA	VAS/GAIS at 10-28 weeks	GAIS W16: PN > HA (*P* = .04)	Elasticity (R2/5/7): PN > HA (*P* < .05) Hydration: PN > HA (*P* = .004) Roughness: PN > HA (*P* = .025) Pores: PN > HA	PN superior skin quality
Yogya et al (2022)^17^ *n* = 30/29	RCT split-face RFMN vs RFMN + PN	Antera 3D wrinkle indentation (6 months)	PN + RFMN: faster improvement (2 months, *P* < .05)	Max depth: similar Patient evaluation: 82% > 25% improvement 51% > 50% improvement	PN + RFMN superior speed
Kim et al (2022)^10^ *n* = 30/28	Case series PN monotherapy	CFGS at 18 weeks	CFGS: 2.77 → 1.84 (*P* < .001)	Antera wrinkle: ↓17% (*P* < .001) Texture: ↓13% (*P* < .001) Pores: ↓16% (*P* < .001) Depression: ↓15% (*P* < .001)	Significant improvement
Jeon et al (2025)^18^ *n* = 15/15	RCT split-face Ionized PN vs conv PN	VAS pain (primary) CFGS (secondary)	VAS: ionized 3.4 vs Conv 5.0 (*P* < .001) CFGS: similar	GAIS: similar	Ionized PN less painful

CFGS, Crow's Feet Grading Scale; Conv PN, conventional (non-ionization-adjusted) polynucleotide formulation; GAIS, Global Aesthetic Improvement Scale; HA, hyaluronic acid; NS, not significant; PCL, polycaprolactone; PN, polynucleotide; RCT, randomized controlled trial; RFMN, radiofrequency microneedling; VAS, visual analog scale.

Jeong et al, Lee et al, and Choi et al demonstrated the PN efficacy comparable to established biostimulatory fillers, and in some cases, superior skin quality outcomes relative to HA.^9,15,16^ Jeong et al and Choi et al compared a PCL-based filler (DLMR01) with a PN filler (Rejuran) for crow's feet.^15,16^ In the earlier, smaller trial (Jeong et al, *n* = 30), the proportion of sides with at least a 1-grade CFGS improvement at 12 weeks was 48.3% for PCL vs 41.4% for PN, a nonsignificant difference.^15^ The larger subsequent trial (Choi et al, *n* = 215) repeated the comparison and demonstrated noninferiority of PCL to PN with respect to CFGS improvement at rest at Week 12, with absolute improvement rates of 70.2% vs 61.4%, respectively.^16^ Both fillers had favorable safety profiles and similar GAIS scores. It is important to note that in both trials the absolute proportion of sides achieving at least 1-grade CFGS improvement was numerically higher with PCL than with PN, and the larger trial's statistical finding was specifically one of noninferiority of PCL to PN rather than equivalence; these results should not be read as indicating identical efficacy between the 2 agents.

Lee et al compared PN (Rejuran) with a noncrosslinked HA hydrofiller in the periorbital region and showed that both treatments improved crow's feet and infraorbital appearance, but PN had advantages in several biophysical parameters. Investigator-rated GAIS scores were significantly higher on the PN-treated side at Week 16, whereas patient visual analog scale (VAS) ratings of overall improvement did not differ between sides. Skin elasticity measured by Cutometer (R2, R5, R7) improved in both groups, with no significant difference in the pattern of change over time. Hydration measured by Corneometer followed a similar pattern, with PN showing significantly greater improvement when time effects were fixed, and hydration at Week 16 was significantly better than on the HA side. Roughness and pore volume quantified by Antera 3D further favored PN, whereas the dermal thickness showed no consistent between-group differences.^9^

Yogya et al evaluated noninsulated radiofrequency microneedling (RFMN) alone vs RFMN followed by topical PN application (transdermal delivery through the microneedle channels) for periorbital wrinkles. In 29 women with Fitzpatrick III-V skin, wrinkle indentation and maximum depth assessed by Antera 3D improved on both sides over 6-month follow-up, but the PN-treated side showed significantly faster improvement, with earlier reductions in wrinkle indentation evident at the 2-month evaluation and maintained thereafter. Patient self-assessments indicated that at 6 months, the majority of patients reported 25% to 75% improvement in periorbital wrinkles. Mean pain scores during treatment were low (∼2/10), and no serious adverse effects were observed.^17^

Jeon et al focused on tolerability rather than efficacy, comparing a conventional PN filler with an ionization-adjusted PN designed to reduce injection pain without altering concentration or overall composition. Fifteen participants underwent 4 periorbital treatment sessions according to a protocol mirroring standard PN use for crow's feet. Pain scores measured immediately after each session were significantly lower on the ionization-adjusted side (mean VAS ∼3.4) than on the conventional PN side (mean VAS ∼5.0), with a large effect size and consistent advantage across sessions. Erythema, bruising, and transient embossing were all less frequent and shorter in duration with the ionized formulation; for example, embossing occurred in only about 13% of ionized PN procedures vs over half of conventional PN procedures. Both formulations produced similar improvements in CFGS and GAIS over the 12-week follow-up, indicating that the modification did not compromise clinical benefit.^18^

Safety outcomes were favorable across the entire dataset. No study reported any case of vascular occlusion, visual disturbance, infection, granulomatous reaction, permanent scarring, or persistent pigmentary alteration. Adverse events were limited to transient erythema, edema, bruising, local tenderness, and occasional embossing, typically resolving within a few days, with individual AE rates in the studies that reported them generally in the range of roughly 10% to 50% of treated sides for mild erythema/bruising and up to ∼50% for transient embossing with conventional PN formulations (vs ∼13% with an ionization-adjusted formulation). The only comparative AE data came from Jeon et al, which showed that formulation changes can meaningfully reduce immediate postprocedural reactions.^18^

Risk of bias was evaluated using design-appropriate tools in line with the protocol. The overall risk of bias was rated as “some concerns” for 4 of the 5 randomized trials and “serious” for the single nonrandomized case series (Tables 3, 4).

**Table 3.** Risk of Bias Assessment of Randomized Controlled Trials (Cochrane RoB 2 Tool)

Author (year)	D1 randomization	D2 deviations from intended intervention	D3 missing outcome data	D4 measurement of outcome	D5 selection of reported result	Other	Overall
Jeong et al (2020)^15^	?	+	+	+	?	?	?
Choi et al (2025)^16^	?	+	+	+	?	?	?
Lee et al, (2020)	+	+	+	+	?	?	?
Yogya et al, (2022)^17^	?	+	+	+	?	?	?
Jeon et al (2025)^18^	?	+	+	?	?	?	?

Assessment according to the Cochrane RoB 2 tool, presented in the following format. (+) = low risk of bias; (?) = some concerns (moderate risk of bias); (x) = high/serious risk of bias; (!) = critical risk of bias. D1 to D5 correspond to the RoB 2 bias domains: randomization process; deviations from intended interventions; missing outcome data; measurement of the outcome; and selection of the reported result.

**Table 4.** Risk of Bias Assessment of the Nonrandomized Case Series (ROBINS-I Framework)

Author/year	Confounding	Selection of participants	Classification of interventions	Deviations from intended intervention	Missing data	Measurement of outcome	Selection of reported result	Overall
Kim et al (2022)^10^	x	x	+	+	+	?	?	x

Assessment according to the ROBINS-I framework, presented in the following format. (+) = low risk of bias; (?) = moderate risk of bias; (x) = serious risk of bias; (!) = critical risk of bias.

## DISCUSSION

The available evidence suggests that PN-based injectables are a clinically effective modality for periorbital rejuvenation, particularly for crow's feet, with an excellent short-term safety profile. Albeit no concerns have been raised on the long-term safety of PNs, none of the selected studies have addressed this point. Across 5 randomized split-face trials and the prospective series, PN formulations—most commonly 20 mg/mL salmon-derived DNA fragments—produced consistent improvements in static and dynamic lateral canthal lines, with between 40% and 70% of treated sides achieving at least a 1-grade reduction in crow's feet severity at 12 weeks and significant improvements in objective wrinkle metrics and dermal microrelief sustained to 18 to 28 weeks in the longest studies.^9,10,15-18^ This range spans trials of different scale and design rather than reflecting a single pooled effect: the smaller, earlier split-face trial (Jeong et al, *n* = 30) reported a 41.4% 1-grade CFGS improvement rate for PN, whereas the larger, more recent trial (Choi et al, *n* = 215) reported 61.4% to 70.2%, with the highest end of the range drawn from an uncontrolled, single-arm case series (Kim et al) prone to overestimation of effect. Differences in sample size, blinding, comparator, assessment time point (rest vs dynamic), and study design account for this heterogeneity, and the 40% to 70% figure should not be interpreted as a single, generalizable effect estimate. These benefits were not limited to wrinkle depth alone. In the PN vs HA trial, PN demonstrated superior improvement in skin elasticity, hydration, surface roughness, and pore appearance, and in the Antera-based case series, PN significantly improved wrinkle, texture, pore, and depression scores. This aligns with the conceptualization of PN as a dermal biostimulator promoting fibroblast activity and extracellular matrix remodeling rather than simply filling space.^5^ This mechanism is distinct from PDRN, whose effects are mediated through adenosine A_2A_ receptor signaling rather than direct fibroblast biostimulation; hence, the 2 agents should not be considered interchangeable in efficacy discussions.^4^

The comparative evidence indicates that PN was noninferior to PCL-based biostimulatory fillers for crow's feet correction, rather than equivalent to them. In both the seminal 30-patient split-face RCT (Jeong et al) and the much larger 215-patient trial (Choi et al), PCL (DLMR01) scores were numerically higher than PN (Rejuran) scores, and PCL and PN yielded similarly high proportions of sides with at least 1-grade CFGS improvement at 12 weeks, with the larger trial formally demonstrating noninferiority of PCL to PN.^15,16^ These findings imply that, within the constraints of short-term follow-up, PN provides wrinkle reduction and subjective aesthetic benefit comparable to other accepted biostimulatory options. It's noteworthy, however, that 1 Rejuran (PN) session achieved comparable efficacy to 1 DLMR01 (experimental PCL) session for crow's feet correction in the largest RCT (Choi et al; *n* = 215). This PCL product is an investigational, microparticle-free formulation, distinct from established commercial PCL fillers like Ellansé (Sinclair Pharma, London, United Kingdom; with microspheres for longer neocollagenesis up to 2-4 years), so its performance specifically for periorbital wrinkles remains unestablished beyond this single trial. Because this comparator differs from commercially available PCL preparations, the comparative findings from this trial should be interpreted with caution and should not be generalized to commercial PCL fillers without further study.

At the same time, the head-to-head PN vs HA data highlight a qualitative difference: PN exerted more pronounced and sustained effects on elasticity, hydration, and fine texture than a noncrosslinked HA hydrofiller, which is consistent with its mechanism of stimulating neocollagenesis and dermal reorganization rather than principally acting through immediate volumization.^9,19^

Emerging preclinical evidence further supports PN–HA synergy. Abuyousif et al demonstrated that a PN–HA complex (Innovyal Regenerative Action, Louna Aesthetics, Poisy, France; 5 mg/mL HA + 7.5 mg/mL high-molecular-weight PN) outperformed Profhilo (IBSA [Institut Biochimique SA], Lugano, Switzerland), NCTF 135 HA (Fillmed Laboratoires [formerly Filorga], Paris, France), and Cellbooster Glow (Suisselle, Yverdon-les-Bains, Switzerland) in normalized rheology (*G*′ 2.1× higher vs Profhilo), antioxidant capacity, and total collagen induction (72 hours human fibroblast model). In 2 periorbital case reports (monotherapy, 1 mL/side ×2 sessions), PN–HA improved skin brightness/texture without AEs, suggesting multifactorial benefits (regeneration + hydration) for prejuvenation.^20^

This has practical implications. For patients with fine rhytides, crepiness, and poor skin quality rather than significant volume loss, PN may be preferable to HA fillers, particularly in the thin periocular dermis where HA can cause overfilling or the Tyndall effect.^8^ In contrast, patients with deep tear trough deformities and structural volume loss are better treated with HA or other volumizers, with PN used as an adjunct to improve overlying skin quality.

Adjunctive use of PN with energy-based devices is also promising. Yogya et al demonstrated that topical PN application through the microchannels created by noninsulated microneedle RF accelerated wrinkle indentation improvement relative to RFMN alone, with benefits evident from 2 months and lasting to 6 months.^17^ This reflects synergy between RF-induced neocollagenesis and PN-mediated fibroblast stimulation and ECM support,^21^ making the combination an attractive option for advanced photoaging or faster results.

Jeon et al show that formulation chemistry can improve procedural tolerability without compromising efficacy. Reduced pain, erythema, bruising, and embossing are clinically relevant given the multiple sessions and dense injections required in the sensitive periocular region. Improved comfort may enhance patient adherence to maintenance treatments. These findings also highlight that PN formulations are not uniform—differences in rheology, pH, osmolarity, and ionization can influence the procedural experience despite similar biological mechanisms.^18^

### Limitations

There are also important limitations. The overall risk of bias was rated as “some concerns” for 4 of the 5 randomized trials and “serious” for the single nonrandomized case series, and this risk-of-bias profile, together with the small number of eligible studies, precluded meta-analysis, and limits confidence in the pooled direction and magnitude of effect. This review is based on only 6 studies, 5 of which were split-face RCTs from a small number of research groups; despite generally low risk of bias in the domains assessed, the modest number and methodological heterogeneity of the included trials (differing comparators, injection protocols, outcome instruments, and follow-up durations) restrict the strength and generalizability of any conclusions. None of the included studies followed patients beyond 6 months, so the durability of PN-induced improvements and optimal retreatment intervals remain uncertain.^6,8,13-16^ Long-term safety of PN injectables warrants particular caution. Because PN is a biologic, salmon-derived DNA fragment product typically administered repeatedly into the thin periocular dermis, short-term trials cannot address the cumulative immunogenic potential of repeated exposure to a xenogeneic biologic, the possibility of delayed granulomatous or nodular reactions after multiple treatment cycles, or the durability vs attenuation of biostimulatory effect with long-term repeated use—none of which have been systematically studied in the included literature. Given the increasing popularity and multi-session nature of PN treatment protocols, dedicated long-term pharmacovigilance and prospective cohorts extending well beyond 6 months are needed before firm conclusions on long-term safety can be drawn. Relatedly, the medium- to long-term efficacy of PN for periorbital rejuvenation is not established by the available data: all included follow-up periods were ≤6 months, and no study assessed durability of effect, need for maintenance treatment, or comparative effectiveness against established modalities beyond this window. Conclusions regarding “efficacy” in this review should therefore be understood as applying specifically to short-term outcomes. No trial directly compared PN plus HA vs HA alone, or PN plus RFMN vs other combinational approaches, limiting information on how PN scores within multimodality treatment algorithms. All participants were recruited from East Asian populations, predominantly with Fitzpatrick III-IV skin; extrapolation to lighter or darker phototypes and different facial morphologies must be done cautiously. The search strategy for this review was also limited to MEDLINE and Embase; Cochrane CENTRAL and regional/Asian-language databases (eg, KoreaMed) were not searched, which may have omitted additional Korean- or Thai-language studies relevant to this predominantly East Asian evidence base. Furthermore, none of the included studies reported cost-effectiveness analyses or formal quality-of-life outcomes. Further, the risk-of-bias assessment moderates the strength of the conclusions that can be drawn.

## CONCLUSIONS

Findings from 6 studies (5 independent split-face RCTs and 1 prospective case series) suggest short-term efficacy and a favorable safety profile of PN for periorbital rejuvenation, specifically in East Asian, predominantly female cohorts with Fitzpatrick III-V skin, with safety established only to 6 months and no data on medium- or long-term durability. Direct comparisons across the studies reviewed here are limited by heterogeneity in comparator, sample size, assessment methodology, and follow-up duration. Conclusions about relative efficacy between agents, and any generalization beyond the populations and timeframes studied, should be drawn with caution. Within these limits, PN injectables may represent a reasonable option for patients with mild-to-moderate crow's feet and textural change seeking gradual improvement through a short treatment series, with PN considered an adjunct rather than a substitute for volumizing fillers in cases of significant structural volume loss. Future studies should focus on larger, multicenter, ethnically and phenotypically diverse RCTs with ≥12-month follow-up, standardized outcomes, and head-to-head comparisons with HA fillers, PCL, and combination regimens before these findings can be considered generalizable.

## Supplementary Material

ojag200_Supplementary_Data

## References

[1] Lambros V . Observations on periorbital and midface aging. Plast Reconstr Surg. 2007;120:1367–1376. doi: 10.1097/01.prs.0000279348.09156.c3 17898614

[2] Kumar N, de Almeida GOO, Luthra A, Kashif SA, Dahan S, Carruthers JDA. Use of high-intensity focused ultrasound in the periorbital rejuvenation: a systematic review. Aesthet Surg J Open Forum. 2026;8:ojag099. doi: 10.1093/asjof/ojag099 PMC13367436 42453157

[3] Yang F, Ji Z, Peng L, et al Efficacy, safety and complications of autologous fat grafting to the eyelids and periorbital area: a systematic review and meta-analysis. PLoS One. 2021;16:e0248505. doi: 10.1371/journal.pone.0248505 PMC8016360 33793573

[4] Marques C, Porcello A, Cerrano M, et al From polydeoxyribonucleotides (PDRNs) to polynucleotides (PNs): bridging the gap between scientific definitions, molecular insights, and clinical applications of multifunctional biomolecules. Biomolecules. 2025;15:148. doi: 10.3390/biom15010148 PMC11764130 39858543

[5] Lee KWA, Chan KWL, Lee A, et al Polynucleotides in aesthetic medicine: a review of current practices and perceived effectiveness. Int J Mol Sci. 2024;25:8224. doi: 10.3390/ijms25158224 PMC11311621 39125793

[6] Colangelo MT, Galli C, Guizzardi S. Polydeoxyribonucleotide regulation of inflammation. Adv Wound Care (New Rochelle). 2020;9:576–589. doi: 10.1089/wound.2019.1031

[7] Lampridou S, Bassett S, Cavallini M, Christopoulos G. The effectiveness of polynucleotides in esthetic medicine: a systematic review. J Cosmet Dermatol. 2025;24:e16721. doi: 10.1111/jocd.16721. PMC11845969 39645667

[8] Zein M, Tie-Shue R, Pirakitikulr N, Lee WW. Complications after cosmetic periocular filler: prevention and management. Plast Aesthet Res. 2020;7:44. doi: 10.20517/2347-9264.2020.133 PMC7583139 33102629

[9] Lee YJ, Kim HT, Lee YJ, et al Comparison of the effects of polynucleotide and hyaluronic acid fillers on periocular rejuvenation: a randomized, double-blind, split-face trial. J Dermatolog Treat. 2022;33:254–260. doi: 10.1080/09546634.2020.1748857 32248707

[10] Kim JH, Kim ES, Kim SW, Hong SP, Kim J. Effects of polynucleotide dermal filler in the correction of crow's feet using an Antera three-dimensional camera. Aesthetic Plast Surg. 2022;46:1902–1909. doi: 10.1007/s00266-022-02832-8 35357558

[11] Shamseer L, Moher D, Clarke M, et al Preferred reporting items for systematic review and meta-analysis protocols (PRISMA-P) 2015 statement. Syst Rev. 2015;4:1. doi: 10.1186/2046-4053-4-1 PMC4320440 25554246

[12] Page MJ, McKenzie JE, Bossuyt PM, et al The PRISMA 2020 statement: an updated guideline for reporting systematic reviews. BMJ. 2021;372:n71. doi: 10.1136/bmj.n71 PMC8005924 33782057

[13] Sterne JAC, Savović J, Page MJ, et al Rob 2: a revised tool for assessing risk of bias in randomised trials. BMJ. 2019;366:l4898. doi: 10.1136/bmj.l4898 31462531

[14] Sterne JAC, Hernán MA, Reeves BC, et al ROBINS-I: a tool for assessing risk of bias in non-randomised studies of interventions. BMJ. 2016;355:i4919. doi: 10.1136/bmj.i4919 PMC5062054 27733354

[15] Jeong GJ, Ahn GR, Park SJ, Hong JY, Kim BJ. A randomized, patient/evaluator-blinded, split-face study to compare the efficacy and safety of polycaprolactone and polynucleotide fillers in the correction of crow's feet: the latest biostimulatory dermal filler for crow's feet. J Cosmet Dermatol. 2020;19:1593–1599. doi: 10.1111/jocd.13199 31680395

[16] Choi SY, Koh YG, Yoo KH, Han HS, Seok J, Kim BJ. A randomized, participant- and evaluator-blinded, matched-pair, prospective study comparing the safety and efficacy between polycaprolactone and polynucleotide fillers in the correction of crow's feet. J Cosmet Dermatol. 2025;24:e16576. doi: 10.1111/jocd.16576 PMC11754925 39313949

[17] Yogya Y, Wanitphakdeedecha R, Wongdama S, Nanchaipruek Y, Yan C, Rakchart S. Efficacy and safety of using noninsulated microneedle radiofrequency alone versus in combination with polynucleotides for treatment of periorbital wrinkles. Dermatol Ther (Heidelb). 2022;12:1133–1145. doi: 10.1007/s13555-022-00729-7 PMC9110589 35501660

[18] Jeon W, Park M, Kim SW, Kim J. Efficacy of ionization-adjusted PN filler in minimizing injection pain: a randomized, patient-blinded, split-face study. Aesth Plast Surg. 2025;50:2723–2730. doi: 10.1007/s00266-025-05320-x PMC13102929 41361144

[19] Gulfan MCB, Wanitphakdeedecha R, Wongdama S, Jantanapornchai N, Yan C, Rakchart S. Efficacy and safety of using noninsulated microneedle radiofrequency alone versus in combination with polynucleotides for the treatment of Melasma: a pilot study. Dermatol Ther (Heidelb). 2022;12:1325–1336. doi: 10.1007/s13555-022-00728-8 PMC9209614 35538360

[20] Abuyousif HS, Porcello A, Cerrano M, et al In vitro evaluation and clinical effects of a regenerative complex with non-cross-linked hyaluronic acid and a high-molecular-weight polynucleotide for periorbital treatment. Polymers (Basel). 2025;17:638. doi: 10.3390/polym17050638 PMC11902836 40076130

[21] Xu Y, Zhang Y, Wang H, Tang H, Zeng W, Wen X. Microneedle radiofrequency induces extracellular matrix remodeling through fibroblast activation: a histological study in a porcine model. Surg Med.. 2025;57:528–543. doi: 10.1002/lsm.70033 40444432

