# Supplementary material for: Efficacy of Polynucleotides in Periorbital Rejuvenation: A Systematic Review
Source: Aesthet Surg J Open Forum. 2026 Oct 10;8:ojag200. doi: 10.1093/asjof/ojag200 (PMC13729110; doi:10.1093/asjof/ojag200)
Supplement: Supplementary file 1 [file ojag200_supplementary_data.docx]

**Supplemental Table S1. PRISMA 2020 Checklist**

Checklist for: "Efficacy of Polynucleotides in Periorbital Rejuvenation: A Systematic Review." Completed per Page MJ, McKenzie JE, Bossuyt PM, et al. The PRISMA 2020 statement: an updated guideline for reporting systematic reviews. BMJ. 2021;372:n71.

| **Section/Topic** | **Item #** | **Checklist item** | **Location where item is reported** |
| --- | --- | --- | --- |
| TITLE | 1 | Identify the report as a systematic review. | Title page (p. 1) |
| ABSTRACT | 2 | See the PRISMA 2020 for Abstracts checklist. | Abstract (p. 1) |
| INTRODUCTION - Rationale | 3 | Describe the rationale for the review in the context of existing knowledge. | Introduction, paras 1–2 (p. 1) |
| INTRODUCTION - Objectives | 4 | Provide an explicit statement of the objective(s) or question(s) the review addresses. | Introduction, final para (p. 2–3) |
| METHODS - Eligibility criteria | 5 | Specify the inclusion and exclusion criteria for the review and how studies were grouped for the syntheses. | Methods (p. 3) |
| METHODS - Information sources | 6 | Specify all databases, registers, websites, organisations, reference lists and other sources searched, and the date each was last searched. | Methods (p. 3) |
| METHODS - Search strategy | 7 | Present the full search strategies for all databases, registers and websites, including any filters and limits used. | Methods (p. 3); full search strategy in Supplemental Table S2 |
| METHODS - Selection process | 8 | Specify the methods used to decide whether a study met the inclusion criteria, including how many reviewers screened, whether independently, and if automation was used. | Methods (p. 3) |
| METHODS - Data collection process | 9 | Specify the methods used to collect data, including how many reviewers, whether independently, any processes for obtaining data from study authors, and if automation was used. | Methods (p. 3) |
| METHODS - Data items | 10a/10b | List and define all outcomes and other variables for which data were sought. | Methods (p. 3) |
| METHODS - Study risk of bias assessment | 11 | Specify the methods used to assess risk of bias, including how many reviewers, whether independently, and the tool(s) used. | Methods (p. 3) |
| METHODS - Effect measures | 12 | Specify for each outcome the effect measure(s) used in the synthesis or presentation of results. | Methods (p. 3); Results (p. 4) |
| METHODS - Synthesis methods | 13a-13f | Describe the processes and methods used to decide eligibility for synthesis, prepare data, tabulate/visually display results, synthesize results (including narrative synthesis given no meta-analysis was performed), explore heterogeneity, and assess robustness. | Methods (p. 3); Results (p. 3–6) |
| METHODS - Reporting bias assessment | 14 | Describe methods used to assess risk of bias due to missing results (reporting bias). | Methods (p. 3) |
| METHODS - Certainty assessment | 15 | Describe methods used to assess certainty in the body of evidence for each outcome. | Methods (p. 3); Discussion, Limitations (p. 7–8) |
| RESULTS - Study selection | 16a/16b | Describe results of the search and selection process, ideally with a flow diagram; briefly cite studies excluded and reasons. | Results, opening paragraph (p. 3); Fig 2, PRISMA flow diagram (separate figure file) |
| RESULTS - Study characteristics | 17 | Cite each included study and present its characteristics. | Results (p. 3–4); Table 1 (separate file) |
| RESULTS - Risk of bias in studies | 18 | Present assessments of risk of bias for each included study. | Results (p. 5); Tables 3 and 4 (separate file) |
| RESULTS - Results of individual studies | 19 | For all outcomes, present the summary statistics and effect estimates for each study. | Results (p. 4–5); Table 2 (separate file) |
| RESULTS - Results of syntheses | 20a-20d | Describe characteristics/risk of bias across studies contributing to each synthesis; present results and heterogeneity; present sensitivity analyses. | Results (p. 4–6); Discussion (p. 5–7) – narrative synthesis; no meta-analysis performed |
| RESULTS - Reporting biases | 21 | Present assessment of risk of bias due to missing results for each synthesis. | Discussion, Limitations (p. 7) |
| RESULTS - Certainty of evidence | 22 | Present assessment of certainty in the body of evidence for each outcome. | Discussion, Limitations (p. 7–8) |
| DISCUSSION | 23a-23d | Provide a general interpretation of results, discuss limitations, and discuss implications for practice, policy and future research. | Discussion (p. 5–8) |
| OTHER INFORMATION - Registration and protocol | 24a-24c | Provide registration information, indicate where the protocol can be accessed, and describe/explain amendments. | Abstract (p. 1); Methods (p. 3) (PROSPERO CRD420261288052) |
| OTHER INFORMATION - Support | 25 | Describe sources of financial or non-financial support and the role of funders. | Title page (p. 1) (Funding statement) |
| OTHER INFORMATION - Competing interests | 26 | Declare any competing interests. | Title page (p. 1) (Disclosures/COI statement) |
| OTHER INFORMATION - Availability of data, code and other materials | 27 | Report which of the following are publicly available: template data collection forms, extracted data, analytic code, other materials. | Available from the corresponding author on reasonable request |

Supplementary table S2: Search terms

| **#** | **Query** | **Results from 13 Jan 2026** |
| --- | --- | --- |
| 1 | Polynucleotide.tw. | 8,549 |
| 2 | Polynucleotides/ | 8,121 |
| 3 | salmon DNA.tw. | 179 |
| 4 | PN.tw. | 65,642 |
| 5 | skin [booster.tw](http://booster.tw/). | 42 |
| 6 | [periorbital.tw](http://periorbital.tw/). | 18,361 |
| 7 | [peri-orbital.tw](http://peri-orbital.tw/). | 881 |
| 8 | [periocular.tw](http://periocular.tw/). | 9,913 |
| 9 | [peri-ocular.tw](http://peri-ocular.tw/). | 341 |
| 10 | tear [trough.tw](http://trough.tw/). | 872 |
| 11 | [under-eye.tw](http://under-eye.tw/). | 301 |
| 12 | crows [feet.tw](http://feet.tw/). | 1,270 |
| 13 | 1 or 2 or 3 or 4 or 5 | 80,787 |
| 14 | 6 or 7 or 8 or 9 or 10 or 11 or 12 | 30,452 |
| 15 | 13 and 14 | 42 |
